# The Role of Intra-Session Exercise Sequence in the Interference Effect: A Systematic Review with Meta-Analysis

**DOI:** 10.1007/s40279-017-0784-1

**Published:** 2017-09-15

**Authors:** Lee Eddens, Ken van Someren, Glyn Howatson

**Affiliations:** 1 Department of Sport, Exercise and Rehabilitation, Northumbria University, Newcastle upon Tyne, UK; 2 Water Research Group, North West University, Potchefstroom, South Africa

## Abstract

**Background:**

There is a necessity for numerous sports to develop strength and aerobic capacity simultaneously, placing a significant demand upon the practice of effective concurrent training methods. Concurrent training requires the athlete to perform both resistance and endurance exercise within a training plan. This training paradigm has been associated with an ‘interference effect’, with attenuated strength adaptation in comparison to that following isolated resistance training. The effectiveness of the training programme rests on the intricacies of manipulating acute training variables, such as exercise sequence. The research, in the most part, does not provide a clarity of message as to whether intra-session exercise sequence has the potential to exacerbate or mitigate the interference effect associated with concurrent training methods.

**Objective:**

The aim of the systematic review and meta-analysis was to assess whether intra-session concurrent exercise sequence modifies strength-based outcomes associated with the interference effect.

**Methods:**

Ten studies were identified from a systematic review of the literature for the outcomes of lower-body dynamic and static strength, lower-body hypertrophy, maximal aerobic capacity and body fat percentage. Each study examined the effect of intra-session exercise sequence on the specified outcomes, across a prolonged (≥5 weeks) concurrent training programme in healthy adults.

**Results:**

Analysis of pooled data indicated that resistance-endurance exercise sequence had a positive effect for lower-body dynamic strength, in comparison to the alternate sequence (weighted mean difference, 6.91% change; 95% confidence interval 1.96, 11.87 change; *p* = 0.006), with no effect of exercise sequence for lower-body muscle hypertrophy (weighted mean difference, 1.15% change; 95% confidence interval −1.56, 3.87 change; *p* = 0.40), lower-body static strength (weighted mean difference, −0.04% change; 95% confidence interval −3.19, 3.11 change; *p* = 0.98), or the remaining outcomes of maximal aerobic capacity and body fat percentage (*p* > 0.05).

**Conclusion:**

These results indicate that the practice of concurrent training with a resistance followed by an endurance exercise order is beneficial for the outcome of lower-body dynamic strength, while alternating the order of stimuli offers no benefit for training outcomes associated with the interference effect.

## Key Points

**Table:** 

The findings support the practice of a resistance followed by an endurance exercise order for the training outcome of lower-body dynamic strength, during a prolonged (≥5 weeks) concurrent training programme.
There was no support for a given exercise order for the training outcomes of lower-body static strength and muscle hypertrophy. This was true also of maximal aerobic capacity and body fat percentage.
Given that an order effect was only observed for one outcome, in favour of a resistance followed by endurance exercise sequence, this practice may prove to be advantageous for dynamic strength adaptation in individuals who are not able to separate endurance and resistance exercise sessions.

## Introduction

Performance in many professional sports necessitates the athlete to develop muscular strength/power and endurance simultaneously, a dichotomous paradigm that poses a challenge to the optimisation of physiological adaptation. Hickson [1] first reported the ‘interference effect’, i.e. attenuated strength development during a concurrent training model in comparison to that following isolated resistance training. Given the necessity for numerous elite sporting populations to develop strength and aerobic capacity simultaneously, a significant demand has been placed upon the practice of effective concurrent training methods. This demand is also true of recreational exercisers with little time available to train; therefore, completing both types of exercise in a single training session. Concurrent training is defined as the simultaneous integration of both resistance and endurance exercise within a coherent training plan [2]. Establishing effective training methods within a concurrent exercise paradigm requires practitioners to manipulate acute training variables to elicit targeted adaptations for a given training cycle or intervention period. The effectiveness of the training programme therefore rests on the intricacies of manipulating exercise frequency, sequence, intensity, duration and mode.

A meta-analysis by Wilson et al. [3] provided a quantitative approach to investigating the existence of the interference effect, using data from 21 studies. Decrements in adaptation for strength, power and hypertrophy across a training programme were observed in the concurrent groups vs. the resistance training groups; however, these responses were only significantly blunted for power [3]. Conversely, numerous investigations have failed to evidence an interference effect on hypertrophy when comparing concurrent training with resistance training in isolation (see review by Murach and Bagley [4]), while some authors have reported concurrent training to augment muscle growth, but not strength, relative to resistance training alone [5–7]. It is possible to adopt a somewhat myopic view of the concurrent training paradigm, whereby the addition of an endurance stimulus is fatal to strength, hypertrophy and or power. Instead, it is of interest to manipulate training variables, in search of an optimum adaptation for given training load and performance demands.

Investigations to identify mechanisms underpinning the potential interference effect followed the seminal work of Hickson [1]. Residual fatigue was initially theorised to provide a possible explanation for the interference effect because of the decline in strength adaptation occurring in the latter stages of the training programme in the concurrent group, relative to the resistance training group, with a further suggestion that biochemical processes of adaptation might prove a mechanistic reason for these observations [1]. Subsequent work has offered additional possibilities to explain the interference effect. These include sub-optimal intra-muscular glycogen levels post-endurance exercise acting to hinder the quality of subsequent resistance exercise [8], or the capacity for prior endurance exercise to reduce muscular peak torque via a decline in the neural input to the muscle and peripheral contractile mechanisms [9], or indeed, antagonistic processes at the molecular level inhibiting the potential for strength adaptation [2].

Regardless of the mechanism(s) underpinning the interference effect, which seem to be complex and potentially multifactorial, the role of exercise sequence has become a pertinent issue. If the interference effect does exist and athletes are required to train concurrently, it is important for the practitioner to understand the consequences of manipulating the acute training variables of exercise frequency, sequence, intensity and mode. The role of intra-session exercise sequence has been investigated in both acute and chronic scenarios, via molecular signalling response post-exercise [10–12] and monitoring morphological and functional outcomes following training [13–15]. A resistance-endurance exercise order throughout a training programme has been reported to result in increased strength [15–17] and hypertrophy [18], in comparison to the exercise order of endurance preceding resistance training. However, other research has found no advantage to exercise sequence for either strength, hypertrophy or power [14, 19, 20]. Consequently, the research is far from unequivocal and the message for athletes and practitioners is not clear. Additional work is therefore warranted to elucidate exercise sequence effects in a concurrent exercise paradigm.

Given the potential for the order effect to influence an interference effect and the apparent equivocal nature of the body of evidence, the purpose of this work was to examine, with a systematic review and meta-analysis, the role of exercise sequence within the context of the concurrent training interference effect. More specifically, the aim was to determine whether intra-session exercise sequence affects the outcomes of lower-body dynamic and static strength, lower-body power and muscle hypertrophy, maximal aerobic capacity and body fat percentage.

## Methods

This meta-analysis was conducted in accordance with the recommendations and criteria of the Cochrane Collaboration (http://uk.cochrane.org/), in line with the criteria set out in the Preferred Reporting Items for Systematic Reviews and Meta-Analyses statement [21]. The respective procedures were agreed upon ahead of data analysis.

### Criteria for Study Eligibility: Studies and Subjects

To be eligible for inclusion in the original article acquisition, a study had to compare the effects of an exercise sequence within a concurrent training paradigm, on at least one outcome measure of strength, power or hypertrophy. These measures are susceptible to decrements consistent with the concurrent interference effect [1, 3]. Maximal aerobic capacity, defined as maximum oxygen uptake, and body fat percentage were analysed as supplementary outcomes if monitored in studies that qualified for inclusion on the grounds of strength, power or hypertrophy outcomes. To limit the research question to the effect of within-session concurrent exercise sequence, only designs with minimal relief between modes of exercise (≤15 min) were included, thereby excluding designs where both modes of exercise were not performed within close proximity to one another. Search criteria were not restricted on the basis of sex or training status; however, participants had to be reported as healthy and above 16 years of age, forming groups that were of similar training status at the onset of the study (e.g. both trained or untrained).

Studies containing at least two groups, allowing for the comparison of resistance followed by endurance exercise, or vice versa across a prolonged concurrent exercise training programme, were considered for inclusion. The concurrent exercise-training programme had to include at least 2 days of concurrent exercise sessions per week, across a continuous period of at least 5 weeks of training. Outcome measures accepted for lower-body maximal strength capacity were separated into dynamic and static methods. Improvements relating to dynamic strength were limited to measurements of 1-repetition maximum in a variation of the squat, leg press or leg extension exercise. Maximal isometric force recorded against an external resistance was accepted as a measure of static strength. Study inclusion for the outcome of muscle hypertrophy was limited to measurements of a muscle fibre cross-sectional area by histochemical analysis or measures of whole muscle volume or thickness by magnetic resonance imaging or ultrasound, respectively. Maximal immediate power, expressed in a dynamic movement (e.g. counter-movement jump) was required for inclusion on the basis of power. Aerobic capacity was determined by measurement of peak oxygen consumption during, or maximal workload at the end of, an incremental test to volitional exhaustion. Body fat percentage measures were limited to dual-energy X-ray absorptiometry scans or skinfold techniques. All of the targeted outcome measures are reported widely in the literature, with good validity and reliability data [22–24].

### Information Sources and Search Strategy

In line with the Cochrane Collaboration methods, a PICO strategy was used to build search criteria for electronic database searches. PICO relates to the components of population, intervention, comparison and outcome. To avoid database bias, a total of four databases were used; PubMed (http://www.ncbi.nlm.nih.gov/pubmed), Web of Science (http://wok.mimas.ac.uk/), MEDLINE (http://ovidsp.tx.ovid.com) and Science Direct (http://www.sciencedirect.com/). Database searches were performed in February 2016 and limited to the year 1980 onwards, from the publication of the seminal research relating to the concurrent interference effect [1]. The search strategy is presented in Table 1. Searches for unpublished data were completed on trial registries (http://clinicaltrials.gov/ and http://www.clinicaltrialsregister.eu/). Following this, a primary exclusion was conducted based on an appraisal of study abstracts. In addition, supplementary searches were conducted by consulting key reviews in the field, along with a search of the reference lists in all articles found. A secondary exclusion was then conducted based on a review of full-text articles. Only studies reported in English-language sources were included. Articles were also scanned for possible duplication and contact with authors was made where duplication of results was possible.

**Table 1:** PubMed search strategy performed on 5 February, 2016

Concept search strategy	Line no.	Entry
Trained/untrained	1	Train*
	2	Athlete*
	3	Recreational exercise*
	4	“Athletic performance/physiology” [Mesh]
	5	1 or 2 or 3 or 4
Concurrent exercise	6	Concurrent exercise*
	7	Concurrent training*
	8	Combined training*
	9	6 or 7 or 8
RCTs	10	Randomized
	11	Randomly
	12	Control*
	13	Training study
	14	10 or 11 or 12 or 13
	15	5 and 9 and 14

Results limited to 1980 onwards (to account for seminal research)

*RCTs* randomised controlled trials

### Study Selection and Data Processing

A study was excluded if it compared exercise sequence without controlling for relief between different modes of exercise, or imposed a design presenting nutritional imbalances between groups. Data processing required a percentage mean change [±standard deviation (SD)] for both groups following the intervention period. These data were acquired for each outcome measure of interest, along with subject numbers in each experimental group. The primary author (LE) read the full text of all studies selected for entry into the meta-analysis (18 studies) and independently extracted data into a pilot form, where data were reported appropriately (3 studies). The primary author (LE) then contacted researchers from the remaining studies to request the data in the required format, or to ask for further information on study methods. A second author (GH) was responsible for independent appraisal of study selection and data extraction, with any disagreements referred to a third author (KvS) for a final decision. To provide an indication of whether publication bias was present, funnel plot symmetry was assessed for each outcome measure, while the *I* ^2^ statistic was used to quantify inconsistency across studies.

The mean difference was calculated for each study by comparison of mean percentage change from pre- to post-intervention for each experimental group, i.e. resistance followed by endurance exercise or endurance followed by resistance exercise. The SD of the mean change was also collected to enable the generation of forest plots with study-specific point estimates and respective 95% confidence intervals. The analyses of the pooled data were conducted with a fixed-effects model, where weighting was attributed based on inverse variance. Where the *I* ^2^ statistic was ≥50%, a random-effects model was used to account for the high heterogeneity. All calculations were performed using Review Manager (RevMan, Version 5.3; The Nordic Cochrane Centre, The Cochrane Collaboration, Copenhagen, 2014).

### Quality Assessment

The quantitative assessment tool ‘QualSyst’ was used to assess methodological quality [37]. The tool contains 14 items scored depending on the degree to which specific criteria were met (yes = 2, partial = 1, no = 0), while items that were not applicable were marked ‘NA’. A summary score was calculated for individual studies by summing the total score obtained across relevant items and dividing it by the total possible score. A score of ≥75, 55–75 and ≤55% indicated strong, moderate and weak quality, respectively. Two reviewers (LE and GH) independently performed quality assessments, with any disagreements referred to a third author (KvS) for consensus.

## Results

The database searches using PubMed, Web of Science, MEDLINE and Science Direct returned 129, 99, 90 and 64 articles, respectively, with 81 full texts retrieved and 18 studies selected for possible entry into the meta-analysis. Despite the common theme of observing the effect of manipulating exercise sequence within a concurrent training programme, the included studies had slightly different aims. Three studies focussed exclusively on applied training outcomes [14, 19, 25], while four studies were focussed on the neuromuscular adaptations to training [16–18, 26], with the remaining studies aiming to investigate the response in hormone concentrations [27], vascular function [15] or gene expression [20], following alternate concurrent exercise sequences.

**Fig. 1:**
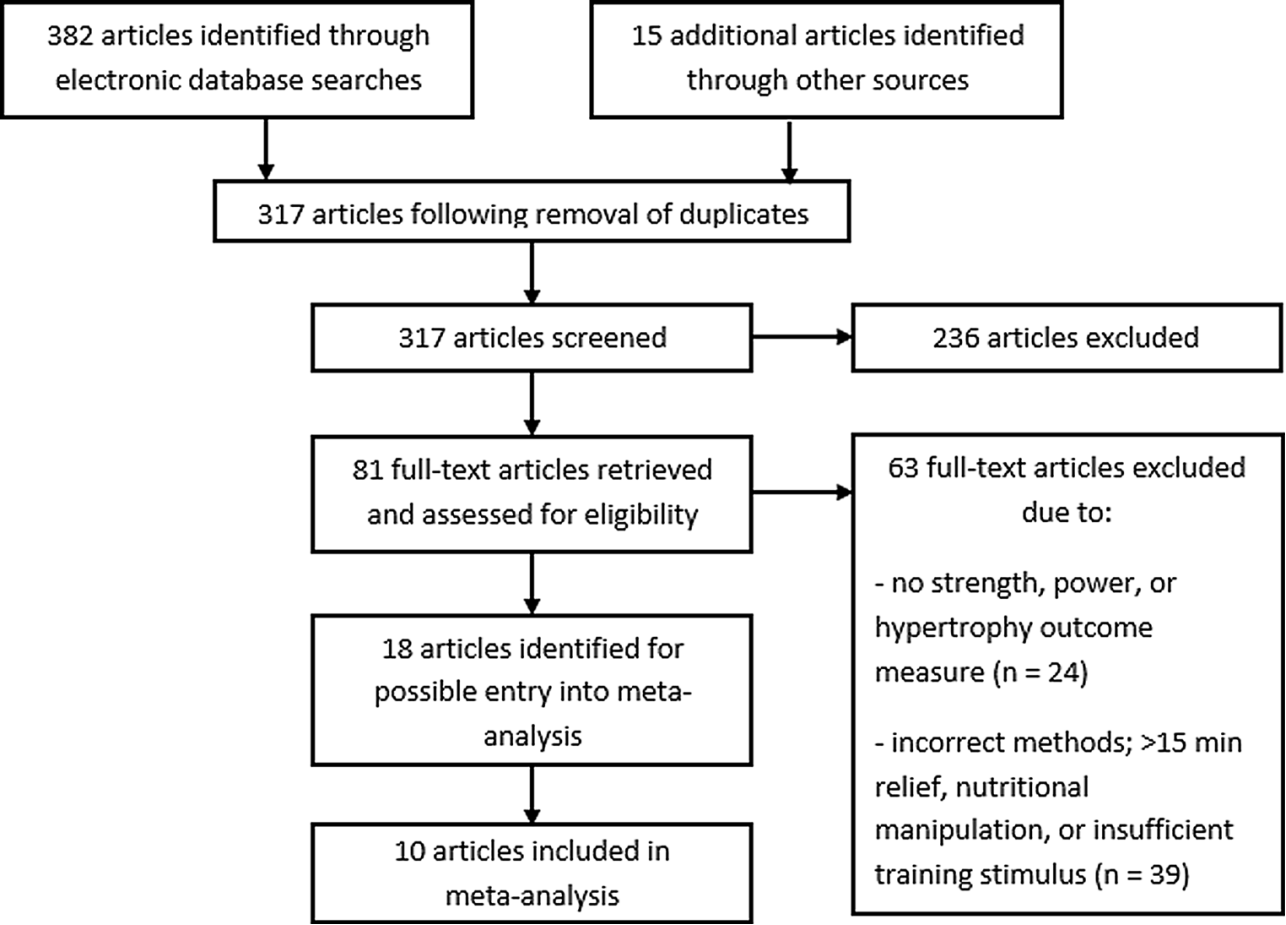
Flow diagram of study screening process

The call to authors resulted in confirmation of duplicated results (three studies), ineligible research (two studies), destroyed data (one study) and non-responders (two studies), leaving ten studies suitable for inclusion in the meta-analysis. Hence, a total of ten studies, including results from 20 groups, met all of the inclusion criteria and were included in the review (Fig. 1). This incorporated a total population size of 227 subjects for lower-body dynamic strength, 155 subjects for lower-body static strength, 137 subjects for lower-body muscle hypertrophy, 167 subjects for body fat percentage and 184 subjects for maximal aerobic capacity. The publication dates ranged from 1993 to 2016. Quality assessments of these ten studies determined that seven were of strong quality and three were of moderate quality (Table 2).

**Table 2:** Quality assessment ‘QualSyst’ [37]

Study	Question described	Appropriate study design	Appropriate subject selection	Characteristics described	Random allocation	Investigator blinded	Subject blinded	Outcome measures well defined and robust to bias	Sample size appropriate	Analytic methods well described	Estimate of variance reported	Controlled for confounding	Results reported in detail	Conclusion supported by results	Rating
Cadore et al. [16]	2	2	2	2	1	2	NA	2	2	2	2	2	2	2	Strong
Chtara et al. [19]	2	2	2	1	0	0	NA	2	1	2	1	1	2	2	Moderate
Collins and Snow [25]	2	2	1	2	1	0	NA	1	2	2	2	1	2	2	Strong
Eklund et al. [26]	2	2	2	2	0	0	NA	2	2	2	2	2	2	2	Strong
Eklund et al. [27]	2	2	2	2	0	0	NA	1	2	2	2	2	2	2	Strong
MacNeil et al. [20]	2	2	2	2	1	0	NA	1	1	2	1	1	2	2	Moderate
McGawley and Andersson [14]	2	1	2	2	0	0	NA	1	1	1	2	1	2	1	Moderate
Okamoto et al. [15]	2	2	2	2	1	1	NA	1	2	1	1	1	2	2	Strong
Pinto et al. [18]	2	2	2	2	1	2	NA	1	2	2	2	2	2	2	Strong
Pinto et al. [17]	2	2	2	2	1	2	NA	1	2	2	2	2	2	2	Strong

*NA* not applicable, *2* yes, *1* partial, *0* no, *strong* strong quality (≥75%), *moderate* moderate quality (55–75%), *weak* weak quality (≤55%)

### Study Characteristics

Data were sourced from a total of 245 subjects with a mean age of 31 ± 16 years, where two studies observed older (aged >55 years) subjects and eight studies were conducted in younger (aged <30 years) subjects (Table 3). Of the ten studies, one study was conducted in professional athletes, three studies observed recreationally active cohorts, while six studies were conducted in untrained subjects. The 20 groups in the analysis comprised male (8 groups), female (6 groups) and mixed (6 groups) cohorts.

**Table 3:** Characteristics of the individual studies included in the meta-analysis

Study	Mean age (years)	Training status	Concurrent training details					
			Study length (weeks)	Training frequency	Relief duration (min)	RES volume range sets × reps (intensity)	END modality	END duration (intensity)
Cadore et al. [16]	65	Untrained	12	3 days/week	<10	2–3 × 6–20 (48–93% 1-RM)	Cycling	20–30 min (80–95% HR_VT_)
Chtara et al. [19]	21	Recreational	12	2 days/week	<15	4–5 × 5–32 (circuit training)	Running	Variable ($\dot{V}{\text{O}}_{2\hbox{max} }$ intervals)
Collins and Snow [25]	22	Untrained	7	3 days/week	None	2 × 3–12 (50–90% 1-RM)	Running	25 min (60–90% HRR)
Eklund et al. [26]	29	Recreational	24	2 days/week to 5 days/2 weeks	<10	2–5 × 3–20 (40–95% 1-RM)	Cycling	25–50 min (~AT/ at or >AnT)
Eklund et al. [27]	29	Recreational	24	2 days/week to 5 days/2 weeks	<10	2–5 × 3–20 (40–95% 1-RM)	Cycling	30–50 min (~AT/ at or >AnT)
MacNeil et al. [20]	20	Untrained	6	3 days/week	None	3 × 10 (65–80% 1-RM)	Cycling	22.5 min (65–75% $\dot{V}{\text{O}}_{2\hbox{max} }$)
McGawley and Andersson [14]	23	Trained	5	3 days/week	<5	2–3 × 4–20 (75–90% 1-RM)	Running (football-specific)	30 min (90–95% HR_max_ intervals)
Okamoto et al. [15]	18	Untrained	8	2 days/week	None	5 × 8–10 (80% 1-RM)	Running	20 min (60% THR)
Pinto et al. [18]	25	Untrained	12	2 days/week	None	3–6 × 10–20 s (maximal effort)	Water-based	18–36 min (HR_VT2_)
Pinto et al. [17]	57	Untrained	12	2 days/week	None	3–6 × 10–20 s (maximal effort)	Water-based	18–36 min (HR_VT2_)

*AnT* anaerobic threshold, *AT* aerobic threshold, *END* endurance training, *HR*_*max*_ maximum heart rate, *HRR* heart rate reserve, *HR*_*VT*_ heart rate at ventilatory threshold, *HR*_*VT2*_ heart rate at second ventilatory threshold, *THR* targeted heart rate, *reps* repetitions, *RES* resistance training, $\dot{V}O_{2max}$ maximum oxygen uptake, *1-RM* 1-repetition maximum

### Publication Bias and Inconsistency

Effect estimates in the studies with smaller standard errors were closer to the true intervention odds ratio, while symmetry was observed upon visual inspection of each outcome measure funnel plot, indicating no clear evidence for publication bias. However, it must be noted that this provides no guarantee that the analysis is free from publication bias [28]. Of the five outcome measures, calculated *I* ^2^ statistics were as follows: 66% for lower-body dynamic strength, 17% for lower-body static strength, 72% for lower-body muscle hypertrophy, 11% for body fat %, and 0% for maximal aerobic capacity. In line with the Cochrane Collaboration thresholds, values up to 60% represent the possibility of moderate heterogeneity, while values up to 90% may represent substantial heterogeneity.

### Intervention Effects and Pooled Analyses

**Fig. 2:**
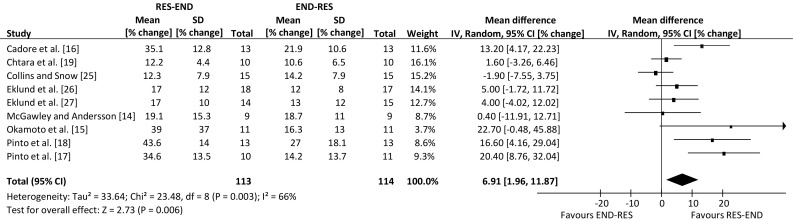
Forest plot of the results of a random-effects meta-analysis shown as pooled mean differences with 95% confidence intervals (CIs) on lower-body dynamic strength (weighted mean difference 6.91%, 95% CI 1.96, 11.87, *p* = 0.006). For each study, the *shaded square* represents the point estimate of the intervention effect. The *horizontal line* joins the lower and upper limits of the 95% CI of this effect. The area of the *shaded square* reflects the relative weight of the study in the meta-analysis. The *shaded diamond* represents the pooled mean difference. *END-RES* endurance training before resistance training, *IV* inverse variance, *RES-END* resistance training before endurance training, *SD* standard deviation

**Fig. 3:**
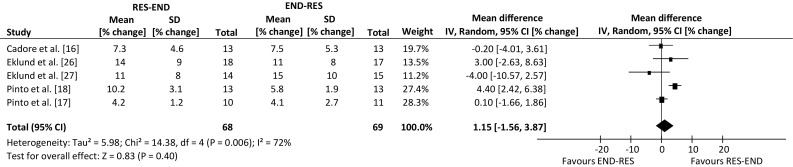
Forest plot of the results of a random-effects meta-analysis shown as pooled mean differences with 95% confidence intervals (CIs) on lower-body muscle hypertrophy (weighted mean difference 1.15%, 95% CI −1.56, 3.87%, *p* = 0.40). For each study, the *shaded square* represents the point estimate of the intervention effect. The *horizontal line* joins the lower and upper limits of the 95% CI of this effect. The area of the *shaded square* reflects the relative weight of the study in the meta-analysis. The *shaded diamond* represents the pooled mean difference. *END-RES* endurance training before resistance training, *IV* inverse variance, *RES-END* resistance training before endurance training, *SD* standard deviation

An overview of the effect from individual studies along with a 95% CI is presented in Table 4. The percentage mean changes following intervention for each of the five outcome measures were individually assessed. Many of the selected publications included further outcome measures, but only those that are relevant to the review have been summarised. The range of mean difference was −1.9 to 22.7% for lower-body dynamic strength, −4.0 to 4.4% for lower-body hypertrophy, −10.0 to 5.5% for lower-body static strength, −5.4 to 1.7% for aerobic capacity and −4.4 to 4.1% for body fat percentage (where a negative value favours endurance-resistance and a positive value favours resistance-endurance exercise sequence). Compared with endurance followed by resistance exercise, performing resistance exercise first enhanced the improvement in lower-body dynamic strength within a prolonged concurrent-type training programme (weighted mean difference, 6.91% change; 95% CI 1.96, 11.87 change; *p* *=* 0.006; Fig. 2). However, exercise sequence had no effect on lower-body muscle hypertrophy, compared with performing endurance exercise first within concurrent training sessions (weighted mean difference, 1.15% change; 95 CI −1.56, 3.87% change; *p* = 0.40; Fig. 3).

**Table 4:** Individual study results included in the meta-analysis

Study	Outcome measures				
	LBDS (95% CI)	LBSS (95% CI)	LBMH (95% CI)	BF% (95% CI)	MAC (95% CI)
Cadore et al. [16]	RE (4.17, 22.23)	RE (−4.19, 8.79)	ER (−4.01, 3.61)	RE (−3.03, 5.23)	ER (−8.77, 6.37)
Chtara et al. [19]	RE (−3.26, 6.46)			RE (−8.44, 8.84)	
Collins and Snow [25]	ER (−7.55, 3.75)				RE (−4.88, 5.28)
Eklund et al. [26]	RE (−1.72, 11.72)	RE (−9.93, 13.93)	RE (−2.63, 8.63)		
Eklund et al. [27]	RE (−4.02, 12.02)	ER (−21.37, 1.37)	ER (−10.57, 2.57)	ER (−8.53, 8.23)	ER (−7.85, 5.85)
MacNeil et al. [20]		RE (−5.60, 16.60)		ER (−8.54, 1.54)	ER (−12.91, 10.11)
McGawley and Andersson [14]	RE (−11.91, 12.71)			RE (−7.05, 8.05)	
Okamoto et al. [15]	RE (−0.48, 45.88)			RE (−0.31, 4.11)	
Pinto et al. [18]	RE (4.16, 29.04)	ER (−11.38, 2.98)	RE (2.42, 6.38)		RE (−2.95, 6.35)
Pinto et al. [17]	RE (8.76, 32.04)	RE (−4.16, 6.56)	RE (−1.66, 1.86)		ER (−14.70, 3.90)

*BF%* body fat percentage, *CI* confidence interval, *ER* outcome in the direction of performing endurance exercise first, *LBDS* lower-body dynamic strength, *LBMH* lower-body muscle hypertrophy, *LBSS* lower-body static strength, *MAC* maximal aerobic capacity, *RE* outcome in the direction of performing resistance exercise first

**Fig. 4:**
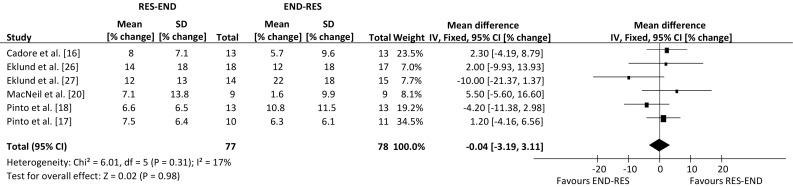
Forest plot of the results of a fixed-effects meta-analysis shown as pooled mean differences with 95% confidence intervals (CIs) on lower-body static strength (weighted mean difference −0.04%, 95% CI −3.19, 3.11, *p* = 0.98). For each study, the *shaded square* represents the point estimate of the intervention effect. The *horizontal line* joins the lower and upper limits of the 95% CI of this effect. The area of the *shaded square* reflects the relative weight of the study in the meta-analysis. The *shaded diamond* represents the pooled mean difference. *END-RES* endurance training before resistance training, *IV* inverse variance, *RES-END* resistance training before endurance training, *SD* standard deviation

**Fig. 5:**
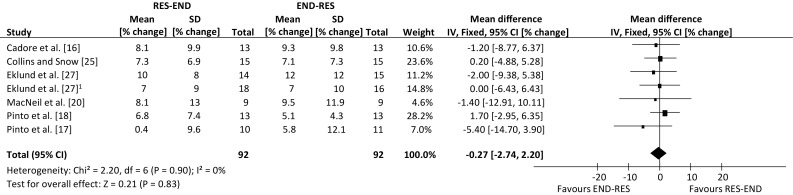
Forest plot of the results of a fixed-effects meta-analysis shown as pooled mean differences with 95% confidence intervals (CIs) on maximal aerobic capacity (weighted mean difference −0.27%, 95% CI −2.74, 2.20, *p* = 0.83). For each study, the *shaded square* represents the point estimate of the intervention effect. The *horizontal line* joins the lower and upper limits of the 95% CI of this effect. The area of the *shaded square* reflects the relative weight of the study in the meta-analysis. The *shaded diamond* represents the pooled mean difference. *END-RES* endurance training before resistance training, *IV* inverse variance, *RES-END* resistance training before endurance training, *SD* standard deviation. ^1^Data collected during study, but obtained through communication with author

**Fig. 6:**
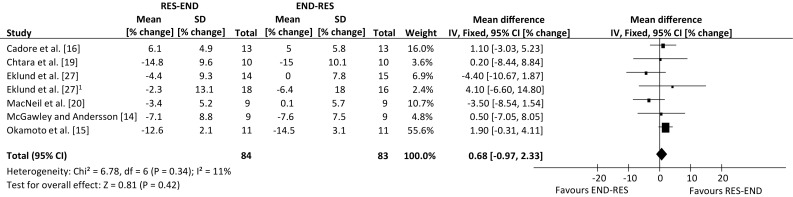
Forest plot of the results of a fixed-effects meta-analysis shown as pooled mean differences with 95% confidence intervals (CIs) on body fat percentage (weighted mean difference 0.68%, 95% CI −0.97, 2.33, *p* = 0.42). For each study, the *shaded square* represents the point estimate of the intervention effect. The *horizontal line* joins the lower and upper limits of the 95% CI of this effect. The area of the *shaded square* reflects the relative weight of the study in the meta-analysis. The *shaded diamond* represents the pooled mean difference. *END-RES* endurance training before resistance training, *IV* inverse variance, *RES-END* resistance training before endurance training, *SD* standard deviation. ^1^Data collected during study, but obtained through communication with author

Exercise sequence had no effect on lower-body static strength (weighted mean difference, −0.04% change; 95% CI −3.19, 3.11% change; *p* = 0.98; Fig. 4). This was also true of maximal aerobic capacity, with improvements following concurrent training not differing between contrasting orders of exercise modes (weighted mean difference, −0.27% change; 95% CI −2.74, 2.20% change; *p* = 0.83; Fig. 5). Finally, performing endurance exercise prior to resistance exercise had no significant effect on body fat percentage, compared with performing resistance exercise first throughout a concurrent training programme (weighted mean difference, 0.68% change; 95% CI −0.97, 2.33% change; *p* = 0.42; Fig. 6). There were not enough data to compare the effects of exercise sequence on lower-body power (2 studies).

## Discussion

This is the first meta-analytic review to assess the role of exercise sequence within the context of the concurrent training interference effect. Pooled estimates revealed that intra-session exercise sequence during a prolonged (≥5 weeks) concurrent training programme significantly affected the improvements in lower-body dynamic strength, with a resistance followed by endurance exercise order superior to the alternative sequence. Meanwhile, the training outcomes of lower-body static strength and muscle hypertrophy were not significantly affected by intra-session sequencing of the exercise mode. Finally, maximal aerobic capacity and body fat percentage, outcomes not associated with concurrent training interference [3], were unaffected by intra-session exercise sequence.

Evidence exists to support the concurrent interference effect, which consists of decrements in strength-based outcomes when practising this type of training relative to resistance training in isolation [3]. As such, it is of interest to observe whether the manipulation of exercise sequence can play a role in mitigating, or indeed exacerbating, this phenomenon. This was true of lower-body dynamic strength, with resistance-endurance exercise sequence proving superior to the alternative order. Previous research suggests that a resistance followed by endurance exercise sequence is beneficial when prioritising strength-based outcomes [15–17], supporting the finding for lower-body dynamic strength. Interestingly, Hickson [1] failed to report exercise sequence of the concurrent training group. This lack of reporting prevents confirmation as to whether the primary finding of this research would act to mitigate or exacerbate the interference effect associated with concurrent training in this original research.

When contextualising the findings of this research, it is important to understand the factors governing adaptation to contrasting types of maximal efforts. The concept of training specificity is well established, whereby resistance training consisting of dynamic contractions results in greater gains during isotonic vs. isometric contractions [29], and hence a degree of contraction-type specificity. Further, adaptation is specific to the contraction velocity of the training stimulus; Kanehisa and Miyashita [26] reported maximal torques at isokinetic speeds, which coincided with the contraction velocity region of the training stimulus. We observed that strength adaptation, following a concurrent training programme, was only susceptible to modification from exercise sequence during dynamic and not static contractions. The greater increase in dynamic vs. static strength, irrespective of exercise sequence, is likely explained by the dynamic training methods of the included studies. However, the effect of training specificity fails to explain why dynamic strength was the only outcome to be modified by intra-session exercise sequence.

There is support from research investigating the order effect that the resistance stimulus should precede endurance exercise, given that residual fatigue from alternate exercise sequence has been suggested to negatively affect the training-induced strength gains [16, 17, 31]. The primary finding of this meta-analysis therefore supports this premise, given that lower-body dynamic strength adaptation was improved following resistance-endurance exercise sequence. What is less clear is why this outcome was modified by exercise order. It is possible that the observed order effect is explained by residual fatigue, with the stress of the preceding endurance stimulus acting to hinder the quality of the resistance session. Indeed, Lepers et al. [9] reported that 2 h of cycling at 65% maximal aerobic power reduced muscular peak torque by 14% in well-trained cyclists, with these outcomes ascribed to a decline in the neural input to the muscle and peripheral mechanisms. Cadore et al. [16] postulated that greater adaptation in lower-body dynamic 1-repetition maximum with resistance-endurance exercise sequence might be attributed to improved neuromuscular economy, with improvements in strength and reduced electromyographic activity for a given load. A suggested role for adjustments in the nervous system is also supported by Eklund et al. [26], with increased maximal force in combination with an increase in muscle activation in the resistance-endurance training group only. However, if residual fatigue or neuromuscular mechanisms were responsible for the observation that exercise sequence modifies the adaptation in lower-body dynamic strength, it remains to be answered why these factors would not facilitate enhanced lower-body static strength also. The finding that hypertrophy and dynamic strength outcomes were not similarly influenced by exercise sequence has been reported previously in the literature, albeit in an older population [16].

The outcome of power is reported to be most susceptible to interference from concurrent training methods [3], suggesting that velocity of contraction during maximal efforts may be an important factor. Concurrent training has been reported to attenuate strength adaptation in the high-velocity, low-force region of the force-velocity relationship, relative to resistance training in isolation. Resistance training in isolation improved maximal torque at angular velocities ranging from 0 to 4.19 rad s^−1^, while improvements from concurrent training were limited to the range of 0–1.68 rad s^−1^, despite both groups completing resistance training at an angular velocity of 4.19 rad s^−1^ [32]. This is particularly important in the applied scenario, given that the majority of athletic performances require a limb speed of ≥3.14 rad s^−1^ [30].

The susceptibility of higher velocity actions to the interference effect has further support [6, 33]. Häkkinen et al. [33] reported that concurrent training resulted in attenuated rapid force production, relative to resistance training in isolation, possibly explained by a reduction in rapid voluntary neural activation. If high-velocity contractions against resistance are most affected by the interference effect, it could perhaps be that the order effect would be most apparent during outcomes assessing maximal power, rather than isometric activity. For example, if power is most affected by the addition of endurance stimuli [3], it would seem logical that prioritising the resistance stimulus (with resistance-endurance exercise sequence) would be of greater importance than for an outcome less affected by the opposing endurance stimuli. Unfortunately, there were insufficient data to include power outcomes in this meta-analysis, but generating sufficient data to analyse the order effect on higher velocity maximal effort outcomes would be a pertinent research question to investigate in the future.

The current study provides an overview of the data available on the effect of manipulating exercise sequence on the interference effect. It should be noted that while a meta-analysis does play a role in causal inference, it is not its primary purpose; rather, it provides an assessment of the consistency of results reported at an individual study level, in addition to offering greater precision of the summary effect outcomes [34]. Some of the outcome measures reported had moderate to substantial heterogeneity, indicating a level of inconsistency in the results of individual studies. This could be a representation of the different methods used between individual studies, or indeed, the breadth of the age and training status in the study treatment groups. Despite symmetry in the funnel plot assessment, a publication bias risk was possible because of the inclusion of published articles only in the meta-analysis, with the risk of published articles showing positive findings and the non-publication of research that shows no effect. Further, the search of English-language sources only might have resulted in missed data. Furthermore, the role of exercise intensity within the context of the interference effect is a topical area of research [35, 36]. It is possible that the relatively untrained cohorts included in the meta-analysis were limited by their ability to perform at higher exercise intensities, and the subsequent effect that this could have had on the interference effect or benefit of a given intra-session exercise order is unknown. These are justifiable avenues for future research. Despite the limitations, this meta-analysis provides an assessment of the potential for intra-session exercise sequence to manipulate strength-based outcomes associated with the concurrent training interference effect.

## Conclusions

The findings support the practice of a resistance followed by endurance exercise order for the training outcome of lower-body dynamic strength during a prolonged (≥5 weeks) concurrent training programme. In the majority of athletic scenarios, maximal dynamic strength is of greater importance than static strength, and therefore likely to be more meaningful to the athlete and practitioner. There was no support for a given exercise order for the training outcomes of lower-body static strength and muscle hypertrophy. This was true also of maximal aerobic capacity and body fat percentage. Given that an order effect was only observed for one outcome, it is recommended that individuals limited by time, such that they must train concurrently with minimal relief between modes of exercise, follow a resistance-endurance exercise order. Manipulating acute training variables may help to optimise adaptation. Given the cohorts included in this meta-analysis (and the body of evidence), the conclusions are particularly relevant to recreational exercisers or untrained individuals. Finally, while maximal aerobic capacity and body fat percentage are not associated with concurrent training interference [3], it was important to observe whether they were affected by the order effect. These outcomes are often assessed following endurance interventions and their inclusion in the meta-analysis is a reminder that the concurrent training paradigm is a challenge because of the need for athletes to adapt divergent physiology in parallel.

## References

[1] Hickson RC (1980). Interference of strength development by simultaneously training for strength and endurance. Eur J Appl Physiol Occup Physiol..

[2] Fyfe JJ, Bishop DJ, Stepto NK (2014). Interference between concurrent resistance and endurance exercise: molecular bases and the role of individual training variables. Sports Med.

[3] Wilson JM, Marin PJ, Rhea MR (2012). Concurrent training: a meta-analysis examining interference of aerobic and resistance exercises. J Strength Cond Res.

[4] Murach KA, Bagley JR (2016). Skeletal muscle hypertrophy with concurrent exercise training: contrary evidence for an interference effect. Sports Med.

[5] Lundberg TR, Fernandez-Gonzalo R, Gustafsson T (2013). Aerobic exercise does not compromise muscle hypertrophy response to short-term resistance training. J Appl Physiol 1985.

[6] Lundberg TR, Fernandez-Gonzalo R, Tesch PA (2014). Exercise-induced AMPK activation does not interfere with muscle hypertrophy in response to resistance training in men. J Appl Physiol 1985.

[7] Mikkola J, Rusko H, Izquierdo M (2012). Neuromuscular and cardiovascular adaptations during concurrent strength and endurance training in untrained men. Int J Sports Med.

[8] Jacobs I, Kaiser P, Tesch P (1981). Muscle strength and fatigue after selective glycogen depletion in human skeletal muscle fibers. Eur J Appl Physiol Occup Physiol.

[9] Lepers R, Hausswirth C, Maffiuletti N (2000). Evidence of neuromuscular fatigue after prolonged cycling exercise. Med Sci Sports Exerc.

[10] Coffey VG, Jemiolo B, Edge J (2009). Effect of consecutive repeated sprint and resistance exercise bouts on acute adaptive responses in human skeletal muscle. Am J Physiol Regul Integr Comp Physiol.

[11] Coffey VG, Pilegaard H, Garnham AP (2009). Consecutive bouts of diverse contractile activity alter acute responses in human skeletal muscle. J Appl Physiol.

[12] Jones TW, Walshe IH, Hamilton DL (2016). Signalling responses following varying sequencing of strength and endurance training in a fed state. Int J Sports Physiol Perform.

[13] Davitt PM, Pellegrino JK, Schanzer JR (2014). The effects of a combined resistance training and endurance exercise program in inactive college female subjects: does order matter?. J Strength Cond Res.

[14] McGawley K, Andersson PI (2013). The order of concurrent training does not affect soccer-related performance adaptations. Int J Sports Med.

[15] Okamoto T, Masuhara M, Ikuta K (2007). Combined aerobic and resistance training and vascular function: effect of aerobic exercise before and after resistance training. J Appl Physiol (1985).

[16] Cadore EL, Izquierdo M, Pinto SS (2013). Neuromuscular adaptations to concurrent training in the elderly: effects of intrasession exercise sequence. Age.

[17] Pinto SS, Alberton CL, Bagatini NC (2015). Neuromuscular adaptations to water-based concurrent training in postmenopausal women: effects of intrasession exercise sequence. Age.

[18] Pinto SS, Cadore EL, Alberton CL (2014). Effects of intra-session exercise sequence during water-based concurrent training. Int J Sports Med.

[19] Chtara M, Chaouachi A, Levin GT (2008). Effect of concurrent endurance and circuit resistance training sequence on muscular strength and power development. J Strength Cond Res.

[20] MacNeil LG, Glover E, Bergstra TG (2014). The order of exercise during concurrent training for rehabilitation does not alter acute genetic expression, mitochondrial enzyme activity or improvements in muscle function. PLoS One.

[21] Moher D, Liberati A, Tetzlaff J (2009). Preferred reporting items for systematic reviews and meta-analyses: the PRISMA statement. BMJ.

[22] McCall GE, Byrnes WC, Dickinson A (1996). Muscle fiber hypertrophy, hyperplasia, and capillary density in college men after resistance training. J Appl Physiol.

[23] Reeves ND, Maganaris CN, Narici MV (2004). Ultrasonographic assessment of human skeletal muscle size. Eur J Appl Physiol.

[24] Verdijk LB, van Loon L, Meijer K (2009). One-repetition maximum strength test represents a valid means to assess leg strength in vivo in humans. J Sports Sci.

[25] Collins MA, Snow TK (1993). Are adaptations to combined endurance and strength training affected by the sequence of training?. J Sports Sci.

[26] Eklund D, Pulverenti T, Bankers S (2015). Neuromuscular adaptations to different modes of combined strength and endurance training. Int J Sports Med.

[27] Eklund D, Schumann M, Kraemer WJ (2016). Acute endocrine and force responses and long-term adaptations to same-session combined strength and endurance training in women. J Strength Cond Res.

[28] Lau J, Ioannidis JPA, Terrin N (2006). The case of the misleading funnel plot. BMJ.

[29] Thorstensson A, Karlsson J, Viitasalo JHT (1976). Effect of strength training on EMG of human skeletal muscle. Acta Physiol Scand.

[30] Kanehisa H, Miyashita M (1983). Specificity of velocity in strength training. Eur J Appl Physiol Occup Physiol.

[31] Bell GJ, Petersen SR, Quinney HA (1988). Sequencing of endurance and high-velocity strength training. Can J Sport Sci.

[32] Dudley GA, Djamil R (1985). Incompatibility of endurance-and strength-training modes of exercise. J Appl Physiol 1985.

[33] Häkkinen K, Alen M, Kraemer WJ (2003). Neuromuscular adaptations during concurrent strength and endurance training versus strength training. Eur J Appl Physiol.

[34] Weed DL (2010). Meta-analysis and causal inference: a case study of benzene and non-Hodgkin lymphoma. Ann Epidemiol.

[35] Fyfe JJ, Bishop DJ, Zacharewicz E (2016). Concurrent exercise incorporating high-intensity interval or continuous training modulates mTORC1 signaling and microRNA expression in human skeletal muscle. Am J Physiol Regul Integr Comp Physiol.

[36] Fyfe JJ, Bartlett JD, Hanson ED (2016). Endurance training intensity does not mediate interference to maximal lower-body strength gain during short-term concurrent training. Front Physiol.

[37] Kmet LM, Lee RC, Cook LS (2004). Standard quality assessment criteria for evaluating primary research papers from a variety of fields.

